# The Spatiotemporal Dynamics and Microevolution Events That Favored the Success of the Highly Clonal Multidrug-Resistant Monophasic *Salmonella Typhimurium* Circulating in Europe

**DOI:** 10.3389/fmicb.2021.651124

**Published:** 2021-05-21

**Authors:** Sabrina Cadel-Six, Emeline Cherchame, Pierre-Emmanuel Douarre, Yue Tang, Arnaud Felten, Pauline Barbet, Eva Litrup, Sangeeta Banerji, Sandra Simon, Federique Pasquali, Michèle Gourmelon, Nana Mensah, Maria Borowiak, Michel-Yves Mistou, Liljana Petrovska

**Affiliations:** ^1^Anses, Laboratory for Food Safety, Salmonella and Listeria Unit, Maisons-Alfort, France; ^2^Department of Bacteriology, Animal and Plant Health Agency, Addlestone, United Kingdom; ^3^Department of Bacteria, Parasites and Fungi, Statens Serum Institut, Copenhagen, Denmark; ^4^Robert Koch-Institute, Division of Enteropathogenic Bacteria and Legionella (FG11)/National Reference Centre for Salmonella and Other Bacterial Enteric Pathogens, Wernigerode, Germany; ^5^Department of Agricultural and Food Sciences, Alma Mater Studiorum – University of Bologna, Bologna, Italy; ^6^Ifremer, RBE, SGMM, Health, Environment and Microbiology Laboratory, Plouzané, France; ^7^Department for Biological Safety, German Federal Institute for Risk Assessment (BfR), Berlin, Germany; ^8^Université Paris-Saclay, INRAE, Centre International de Ressource Microbienne (CIRM) MaIAGE, Jouy-en-Josas, France

**Keywords:** epidemic monophasic *Typhimurium*, core and accessory genome phylogenetic analyses, Bayesian analysis, plasmidome, resistome, microevolution European study

## Abstract

The European epidemic monophasic variant of *Salmonella* enterica serovar *Typhimurium* (*S*. 1,4,[5],12:i:-) characterized by the multi *locus* sequence type ST34 and the antimicrobial resistance ASSuT profile has become one of the most common serovars in Europe (EU) and the United States (US). In this study, we reconstructed the time-scaled phylogeny and evolution of this *Salmonella* in Europe. The epidemic *S*. 1,4,[5],12:i:- ST34 emerged in the 1980s by an acquisition of the *Salmonella* Genomic Island (SGI)-4 at the 3′ end of the phenylalanine *phe* tRNA *locus* conferring resistance to copper and arsenic toxicity. Subsequent integration of the Tn21 transposon into the *fljAB locus* gave resistance to mercury toxicity and several classes of antibiotics used in food-producing animals (ASSuT profile). The second step of the evolution occurred in the 1990s, with the integration of mTmV and mTmV-like prophages carrying the *perC* and/or *sopE* genes involved in the ability to reduce nitrates in intestinal contents and facilitate the disruption of the junctions of the host intestinal epithelial cells. Heavy metals are largely used as food supplements or pesticide for cultivation of seeds intended for animal feed so the expansion of the epidemic *S.* 1,4,[5],12:i:- ST34 was strongly related to the multiple-heavy metal resistance acquired by transposons, integrative and conjugative elements and facilitated by the escape until 2011 from the regulatory actions applied in the control of *S.* Typhimurium in Europe. The genomic plasticity of the epidemic *S.* 1,4,[5],12:i:- was demonstrated in our study by the analysis of the plasmidome. We were able to identify plasmids harboring genes mediating resistance to phenicols, colistin, and fluoroquinolone and also describe for the first time in six of the analyzed genomes the presence of two plasmids (pERR1744967-1 and pERR2174855-2) previously described only in strains of enterotoxigenic *Escherichia coli* and *E. fergusonii*.

## Introduction

The majority of the *Salmonella* serovars are motile; they can produce two types of flagellar proteins and switch from one type to the other by the expression regulation of *fliC*, *fljBA*, and *hin* genes. These *Salmonella* strains are called bi-phasic. The monophasic variant of *S*. Typhimurium, whose antigenic formula is *S*. 1,4,[5],12:i:-, lacks expression of phase 2 flagella. This lack of expression is due to partial or complete deletion of the *fljBA locus*, or due to different mutations in *fljA*, *hin* and the promoter controlling the expression of *fljB* and *fliC* (Sun et al., 2020).

Since the first report in the literature of *S*. 1,4,[5],12:i:- isolation in the late 1980s from chicken carcasses in Portugal (Machado and Bernardo, 1990), this serovar has been reported in a variety of countries – Thailand, Spain, Brazil, and United States (US). In less than twenty years, it has become one of the most common serovars in Europe and US (Switt et al., 2009; Wang et al., 2019; Sun et al., 2020). Even if the global epidemics attributed to this serovar between 2009 and 2016 have been principally related to pork sources, *S*. 1,4,[5],12:i:- has colonized all sectors of the food industry and since 2010 has become one of the top serovars causing human infections. From 2011 to 2013, the percentage of non-typhoidal *Salmonella* infections caused by *S*. 1,4,[5],12:i:- has increased from 4.6% (3,739 of 80,782) to 8.6% (6,313 of 73,627) in the European Union member states. Since 2013, this serovar has become the third common cause of non-typhoidal *Salmonella* infections in Europe (European Food Safety Authority [EFSA], and European Centre for Disease Prevention and Control [ECDC], 2014, 2015, 2016, 2017, 2018, 2019). Moreover, while *Salmonella Typhimurium* and monophasic S. 1,4,[5],12:i:- are commonly isolated in animal sources, they can also be present in the environment such as surface waters. For example, they have been isolated in rivers at the outlet of agricultural coastal catchments in France (Rincé et al., 2018). A recent study focusing to evolution of *Salmonella Typhimurium* pointed out a major phylogroup of predominantly Multi Locus Sequence Type (MLST) ST19 and a minor phylogroup of ST36. The major ST19 group revealed two high order clades (α and β) with clade α clustering the epidemic *S*. 1,4,[5],12:i:- ST34 (Bawn et al., 2020).

The epidemic success of this *S.* 1,4,[5],12:i:- ST34, is correlated with selective acquisition of antibiotic (AMR) and heavy metal resistance genes (Petrovska et al., 2016; Biswas et al., 2019; Sun et al., 2020), leading to establishment of multi-drug resistant (MDR) clones (Sun et al., 2020). Tetracycline, wide spectrum penicillins, aminoglycosides and sulfonamides were widely used antibiotics in the pig sector in Europe in the past decades. In addition, the expansion of the ST34 MDR *S.* 1,4,[5],12:i:- was also facilitated in the pig breeding during the 2000s in Europe, as this serovar was not immediately recognized as a variant of *S*. Typhimurium (i.e., one of the most common serovars causing *Salmonella* outbreaks worldwide) and therefore escaped regulatory actions applied to *S.* Typhimurium until 2011.

Altogether, there have been three predominant *S.* 1,4,[5],12:i:- clones circulating in the past two decades – the Spanish, the European and the US clone, two of which are MDR clones. The Spanish clone, characterized by the MLST profile ST19, carries a plasmid conferring resistance to up to seven antimicrobial drugs (i.e., ampicillin, chloramphenicol, gentamicin, streptomycin/spectinomycin, sulfonamides, tetracyclines, and trimethoprim) (Laorden et al., 2010), whereas the European ST34 clone encodes a chromosomal element responsible for the resistance to four antimicrobial drugs (i.e., ampicillin, streptomycin/spectinomycin, sulfonamides, and tetracyclines) (Trupschuch et al., 2010; Petrovska et al., 2016). In contrast, antibiotic resistance has rarely been described in the US clone that emerged around 2004, most likely from an independent event (Hauser et al., 2011).

The Spanish clone was described for the first time in Spain in 1997 (Echeita et al., 1999; Mourao et al., 2014). The European clone was described for the first time in 2005 in Luxembourg (Mossong et al., 2007), and since, has become the main clone in many other countries such as Japan and the United States (Arai et al., 2018). A recent study showed indeed that *S*. 1,4,[5],12:i:- ST34 with antimicrobial resistance ASSuT profile was introduced into swine sector in the US Midwest from EU in 2014 (Elnekave et al., 2018, 2020).

Even if the European clone ST34 is today predominant worldwide, several endemic clones have been reported in several European countries. Retrospective genomic analysis of epidemic strains of *S*. 1,4,[5],12:i:- ST34 from the United Kingdom and Italy, collected between 1993 and 2010, revealed the presence of the genomic island SGI-4, conferring resistance to copper and arsenic (Petrovska et al., 2016). In the United Kingdom, a large number of genomic variations have been described, including the acquisition of a prophage carrying the additional *sopE* gene mediating cellular invasion and disorganization of epithelial cell junctions (Mirold et al., 1999; Petrovska et al., 2016). This prophage was found also in the *S.* 1,4,[5],12:i:- strains circulating in Italy and Germany (Müller et al., 2009; Palma et al., 2018).

In this study, we undertook the phylogenetic analysis of 298 *S*. Typhimurium and *S*. 1,4,[5],12:i:- genomes isolated over eighteen years from human, animal, food, feed and environment from five countries, Denmark, France, Germany, Italy, and the UK. Bayesian analysis of the 205 genomes belonging to the genomic lineage corresponding to the epidemic clone ST34 made it possible to date its appearance in Europe and to date each of the genomic modifications that marked its evolutionary success.

## Materials and Methods

### Strains Dataset

The dataset described in this study was collected in the context of Work Package 4/7 of the Collaborative Management Platform for detection and Analyses of (Re-) emerging and foodborne outbreaks in Europe (COMPARE, Horizon2020 research project grant number 643476) (Munck et al., 2020). This research network aimed to better characterize the *S.* 1,4,[5],12:i:- clones circulating in Europe and develop new methods to analyze/track them. A specific task therein was to retrace the spread of the multidrug-resistant *S.* 1,4,[5],12:i:- and to identify drivers of its specific success. The study of spatiotemporal dynamics of its spread and the genetic basis that favored its high specificity requires data that are: (1) representative of its geographic diversity (multi-countries study), (2) from different origins (human, animal, food, feed and environmental) and from different sampling contexts, (3) related to a period that runs from appearance of *S.* 1,4,[5],12:i:- until today and (4) analyzed using a discriminatory subtyping method. Data collected through this study complies with these requirements and when the choice between strains from the same collection was possible, the RANDBETWEEN function of Excel was used to randomly choose between strains.

The strains used in this study with their metadata (ID, sample name, ID owner, country, collection year, and origin) are listed in the Supplementary Table 1. The overview of the data is presented in Table 1. A representative set of strains of *Salmonella* Typhimurium and the *S.* 1,4,[5],12:i:- from human and different animal, food, feed and environmental sources were collected from five European countries: Denmark (DK), France (FR), Germany (DE), Italy (IT), and the United Kingdom (UK). The strains were collected as part of the national surveillance, monitoring, control programs, research projects and larger surveys conducted between 1992 and 2018. Strains from animals (*n* = 120) and food (*n* = 58) represent 60% of the dataset, human strains (*n* = 37) 12%, environment (*n* = 10), feed (*n* = 2) 4%, and non-specified strains (*n* = 71) represent 24% of the dataset.

**TABLE 1:** Overview of the dataset collected.

**Country**	**Dataset owner**	**Years included**	**Antigenic formula**	**No. of genomes**
UK	APHA/PHE	1992–2018	*S.* 1,4,[5],12:i:-	30
			*S*. Typhimurium	22
DE	RKI	1997–2009	*S.* 1,4,[5],12:i:-	20
			*S*. Typhimurium	4
DE	BfR	2013–2016	*S.* 1,4,[5],12:i:-	46
			*S*. Typhimurium	8
FR	ANSES	2001–2014	*S.* 1,4,[5],12:i:-	43
			*S*. Typhimurium	28
FR	IFREMER	2013–2014	*S.* 1,4,[5],12:i:-	2
			*S*. Typhimurium	1
DK	SSI	2002–2018	*S.* 1,4,[5],12:i:-	45
			*S*. Typhimurium	22
IT	UNIBO	2012–2014	*S.* 1,4,[5],12:i:-	27

*The strains collected belong to eight different collections: APHA (Animal and Plant Health Agency), PHE (Public Health England), RKI (National Reference Center of Salmonella collection of Robert Koch-Institute), BfR (National Reference Laboratory of Salmonella collection of the Federal Institute for Risk Assessment), ANSES (Salmonella Network collection belonging to the Food Safety Laboratory), IFREMER (Health, Environment and Microbiology Laboratory) collection of IFREMER, SSI (Statum Serum Institut), and UNIBO (Department of Agricultural and Food Sciences collection of Alma Mater Studiorum University of Bologna).*

All strains were identified by glass slide agglutination, according to the White-Kauffmann-Le Minor scheme (Grimont and Weill, 2007) and all were confirmed as monophasic variants using the PCR method or *in silico* typing method. Of the initial 320 strains included in the dataset, 96 were Typhimurium (30%) and 224 were *S*. 1,4,[5],12:i:- (70%).

### Sequencing and Assembly

All strains were sequenced using Illumina chemistry producing paired end reads. Twenty-two strains were excluded by the study because of the low genome quality values (data not shown).

Genome assemblies were generated using an in-house workflow called ARTwork (Vila Nova et al., 2019; https://github.com/afelten-Anses/ARtWORK). The raw reads were normalized to 100× with Bbnorm using the *Salmonella Typhimurium*_LT2_AE006468 reference genome. Then, Trimmomatic (Bolger et al., 2014) was used for the trimming step. The applied quality rules were: (1) length of read higher or equal to 50 base pairs (bp) otherwise excluded, (2) phred score per base higher or equal to 30× and (3) filter away adapters based on an internal database with Illumina adapters. FastQC version 0.11.5 was used to control the read quality and ConFindr to identify intra-and cross-species contamination (Low et al., 2019). SPAdes 3.11.05 was used to perform *de novo* assembly. Medusa and Gapcloser were used to optimize and finish the assembly (Kremer et al., 2017). Subsequently, QUAST was used to evaluate the quality of *de novo* assemblies by identifying misassemblies and determining error rates (Gurevich et al., 2013).

Read and assembly quality of the 298 genomes retained in the study is reported in Supplementary Table 1.

### Genomic Analysis

#### Multilocus Sequence Typing (MLST)

All genomes were characterized by *in silico* MLST using seven housekeeping genes *aroC, dnaN, hemD, hisD, purE, sucA*, and *thrA* (Achtman et al., 2012). The seven housekeeping gene sequences for each strain were uploaded to the MLST service of the Center for Genomic Epidemiology (CGE)^1^, which allowed us to determine the sequence type (ST) directly from the read files.

#### Variant Calling (SNPs)

The core genome SNPs and small InDels were detected based on the variant caller HaplotypeCaller implemented in the iVARCall2 workflow (Felten et al., 2017), using *Salmonella* Typhimurium LT2 (NCBI NC_003197.1) as reference genome, and following the best practices proposed by the Genome Analysis ToolKit (GATK) (McKenna et al., 2010). More precisely, secondary alignments around small InDels were performed and duplications were excluded before variant calling analysis via local *de novo* assembly of haplotypes in active regions. The matrices of pairwise SNP differences and pseudogenomes were computed using an in-house Python scripts called “VCFtoMATRIX” and “VCFtoPseudoGenome,” respectively (https://github.com/afelten-Anses/VARtools/tree/master/iVARCall2).

#### Phylogenomic Inference and Calculation of Bootstrap Support

Phylogenomic inferences were performed by maximum likelihood based on pseudogenomes produced by the iVARCall2 workflow and the transversional model TVM+F+I implemented in iQTree program (http://www.iqtree.org/). Node support was evaluated with 1,000 rapid bootstrap inferences (http://www.iqtree.org/). Phylogenetic trees were visualized and annotated using interactive Tree Of Life (iTOL https://itol.embl.de/) (Letunic and Bork, 2016).

#### Identification of Acquired Resistance Genes and Virulence Factors

Whole genomes were screened for the presence/absence of genes mediating antibiotic resistance and virulence using ABRICATE (https://github.com/tseemann/abricate). ABRICATE blast application was used with the ResFinder database available at the Center for Genomic Epidemiology (CGE) (Denmark) and the vfdb database available from the Institute of Pathogen Biology (Bejing, China). The ABICATE outputs show only the genes found on at least one genome of the analyzed panel. A local database of whole genomes was used to search via BLASTN 2.2.28+ (https://blast.ncbi.nlm.nih.gov/Blast.cgi) the regions of the *fljAB*, *thrW*, *yidC* and SGI-4 *loci* as described by Branchu in 2019 (Branchu et al., 2019). The threshold was set at a 90% identity over at least 3/5 of the length of the gene or genomic region.

#### Identification of Prophages

Each of the 298 assembled genomes were analyzed by PHASTER to identify the presence of prophages and their integrase genes (Arndt et al., 2016). Only prophages identified as “intact” or “questionable” were considered. The identity of all intact prophage sequences detected by PHASTER was confirmed by BLAST. The presence of the mTmV prophage encoding *sopE* and *perC* genes was investigated by searching 59 loci of the mTmV prophage of the strain S04698-09 (NZ_LN999997.1) in all the 298 genomes.

#### Identifications of Plasmids and Other Mobile Genetic Elements

All the contigs carrying resistance and virulence genes were further analyzed to characterize the presence of mobile genetic element (MGE). Replicon and MGE typing using PlasmidFinder (Carattoli et al., 2014) and MOB-suite (Robertson and Nash, 2018) used to identify plasmids markers. Putative plasmids were blasted against the NCBI nucleotide archive to identify the closest plasmid neighbor.

The presence of the virulence plasmid encoding the pef, rck, and spv operons was investigated by searching 60 *loci* of the pSLT plasmid of *Salmonella* Typhimurium LT2 (CP014051.2) in all 298 genomes.

### Bayesian Analysis of Molecular Sequences

Bayesian analysis of molecular sequences using Markov chain Monte Carlo (MCMC) was carried out on the 205 genomes of the clade A, the main group of *S*. 1,4,[5],12:i:- ST34 identified. Phylogenomic inferences were performed by maximum likelihood based on pseudogenomes produced by the iVARCall2 workflow and the Kimura model with three substitution types K3Pe+F+I model implemented in the iQtree program (http://www.iqtree.org/). Transition rate (e) and proportion of invariable sites (F+I), as well as convergences of the phylogenomic inferences were checked based on bootstrap analysis. Node support was evaluated with 1,000 rapid bootstrap inferences in the iQtree program (http://www.iqtree.org/). Bayesian phylogenetic analyses were performed using BEAST v. 1.7.5 (Drummond and Rambaut, 2007). The isolation year of each strain was used to establish a temporal framework for constructing phylogenetic relationship among the strains and estimating parameters to describe the evolutionary dynamics of the population (Okoro et al., 2012). Comparisons of different molecular clock models and tree priors were performed. Strict clock and relaxed clock log normal models were implemented with Coalescent Constant Population (CSC) or Coalescent Exponential Population (CEP) priors. Least Squares Dating (LSD) software (To et al., 2016) was also carried out to estimate the rate of evolution.

## Results

### Genetic Diversity and SNP Core Genome Phylogenetic Analysis

**FIGURE 1:**
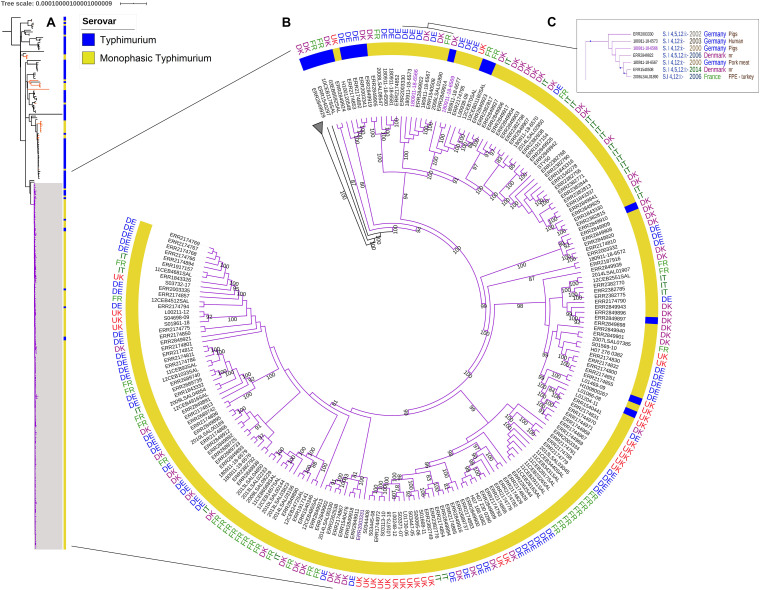
**(A)** SNP core genome phylogenetic tree of 298 *S.* Typhimurium and *S*. 1,4,[5],12:i:- strains. The main clade A including 90% of the *S.* 1,4,[5],12:i:- is in purple. Orange branches indicate the other *S.* 1,4,[5],12:i:- genomes within clade B. **(B)** SNP core genome phylogenetic tree of the clade A. The branch length is ignored. The country are reported for the main *S.* 1,4,[5],12:i:- group in the ring: DE (Germany) in blue, DK (Denmark) in purple, IT (Italy) in dark green, FR (France) in light green, and the UK (United Kingdom) in red. The bootstraps comprised between 80 and 100% are reported. **(C)** The box focus on the group including the oldest strain (180911-18-6566) within the main *S.* 1,4,[5],12:i:- group. In the box, bootstrap values of 100% are represented by a blue triangle.

To investigate the phylogenetic relationship, we constructed a maximum likelihood tree of the variations in the *core* genomes of the 93 *S*. Typhimurium and 205 *S.* 1,4,[5],12:i:- ST34 human, animal, food, feed, and environmental isolates collected in Denmark, France, Germany, Italy, and the United Kingdom between 1992 and 2018. The MLST profile analysis assigned 82 strains to ST19, 204 strains to ST34 and 12 strains to other profiles (Supplementary Table 1). All identified MLST profiles in this study belong to the clade α described by Bawn et al. (2020), with the exception of one strain (ERR2849932) that was assigned to ST313 and belonged to clade B. This strain was used as an outgroup to root the phylogenetic trees. The analyses revealed two distinct clades, a main clade A that included 90% (191/213) of the *S*. 1,4,[5],12:i:- in this study (violet group in Figure 1), and a second clade B, containing mainly (76%) *S*. Typhimurium strains. Bootstraps supporting both clades were 100% (Supplementary Figure 1). The main clade A included 205 strains, 191 *S*. 1,4,[5],12:i:- and 14 *S*. Typhimurium most of which were MLST profile ST34, with the exception of one strain (ERR1540441) that had profile ST4067 (Supplementary Table 1). The average distance within this clade was 41 SNPs with a Standard Deviation (SD) of 9 SNPs. Six *S*. Typhimurium strains (03EB8592SAL, 10CEB1178SAL, ERR2849924, ERR2174853, ERR2174892, and ERR2003341), isolated between 2002 and 2015 in Denmark, France and Germany, from poultry and pig sectors were at the basal node of this main clade of *S*. 1,4,[5],12:i:- strains (Supplementary Figure 1). The two oldest *S*. 1,4,[5],12:i:- ST34 strains within the clade A were 180911-18-6566 and 180911-18-6567 isolated in 2000 in Germany, from pigs and pork, respectively. These strains were closely related to five other *S*. 1,4,[5],12:i:- ST34, two of which (ERR2003330 and 180911-18-6573) were isolated between 2002 and 2003 in Germany, from a pig and a human, respectively and to three strains isolated later – between 2006 and 2014 – in France (2006LSAL01890) and Denmark (ERR2849922 and ERR1540508). This group of seven strains that differed by 28 SNPs (with SD of ± 4 SD) was supported by a bootstrap value of 100% (Figure 1C).

The second clade, clade B, contained the remaining 93 genomes – 71 *S*. Typhimurium and 22 *S*. 1,4,[5],12:i:-, and had an average of 558 ± 108 SNPs. The *S*. Typhimurium strains were isolated between 1992 and 2018 from the five countries (Denmark, France, Germany, Italy, and the United Kingdom). The 22 *S*. 1,4,[5],12:i:- strains isolated between 2001 and 2017 from all five countries were distributed within 12 independent branches (Figure 1 and Supplementary Figure 1, orange branches). Most of the *S.* Typhimurium and all *S*. 1,4,[5],12:i:- isolates in Clade B belonged to ST19 (Supplementary Table 1 and Supplementary Figure 1).

### Genotypic Variation in the *fljAB Locus*

Partial or complete deletions, or insertion sequences in the *flj*AB *locus* have been described as reasons for the lack of expression of phase 2 flagella in the *S*. 1,4,[5],12:i:- strains (Branchu et al., 2019). Of the 213 *S*. 1,4,[5],12:i:- isolates in this study 99% (211/213) contained either Tn21-family composite transposon or type-6 insertion sequences (IS6) at the *fljAB locus* (Supplementary Table 2).

Of these 211 strains, 80% (169/211) contained the Tn21 composite transposon characterized by the presence of mercury resistance cluster (*mer*TABCDE and *mer*R) and also carried one to four genes encoding antibiotic resistance to aminoglycosides (*aph(3”)-Ib_5* and *aph(6)-Ib_1*), beta-lactam (*blaTEM-1B_1*), sulfonamides (*sul2_3*) and tetracycline (*tetB_2*) with 138 strains having the ASSuT profile. The other 20% (42/211) contained in the *fljAB locus* IS6 sequences harboring genes mediating resistance to one to four antibiotic drug families (tetracyclines, sulfonamides, aminoglycosides, and beta-lactams) – but lacked the mercury resistance cluster. Interestingly, of the 169 strains containing the mercury resistance cluster, 158 belonged to the clade A and 11 to the clade B (Supplementary Table 2).

Insertional sequences (Tn21 and IS6) or deletions in the *fljAB* and *hin* region were present in 13 of the 14 *S*. Typhimurium genomes in the clade A. Only the genome ERR2849924 isolated in Denmark in 2002, had neither gene deletions nor insertions in *fljAB locus*. This genome was positioned in the deepest branch of the clade A, with 10CEB1178SAL and 03CEB8592SAL strains isolated in France in 2003 and 2010, respectively (Figure 1B). SeqSero tool analysis (https://cge.cbs.dtu.dk/services/SeqSero/) confirmed the Typhimurium prediction for the ERR2849924 strain (data not shown).

### Acquisition of the *Salmonella* Genomic Island (SGI)-4

The *Salmonella* Genomic Island 4 (SGI-4) was described as one of the microevolutionary events characteristic of the *S*. 1,4,[5],12:i:- ST34 clade that was absent from other *S.* Typhimurium strains in the UK and Italy (Petrovska et al., 2016). Of the 298 genomes analyzed in this study, 192 (64%) carried the SGI-4 with arsenic and copper resistance gene clusters, all of which belonged to clade A (Supplementary Table 3). The oldest strains carrying the SGI-4 were 180911-18-6566 and 180911-18-6567 isolated in 2000 in Germany from pigs and pork, respectively (Figure 1 and Supplementary Figure 1). The analysis of the flanking regions of the SGI-4 in the 192 genomes revealed that this conjugative element was integrated in the genome at the 3′ end of the phenylalanine tRNA-phe *locus*, as previously described by Branchu et al. (2019). This particular tRNA *locus* is present in two copies in the LT2 genome at 3.2 and 4.6 Mb and we found that the SGI-4 was integrated in both sites but preferentially in the tRNA-phe *locus* at 4.6Mb (Supplementary Table 4).

### Phage Mediated Acquisition of *sopE* and *perC* genes in the European *S.* 1,4,[5],12:i:- ST34 Isolates

The temperate prophages mTmV and mTmV2, carrying virulence *sopE* gene described in *S.* 1,4,[5],12:i:- ST34 (Petrovska et al., 2016; Tassinari et al., 2020) was also shown to lead to a clonal expansion of this clone in the UK (Tassinari et al., 2020).

**FIGURE 2:**
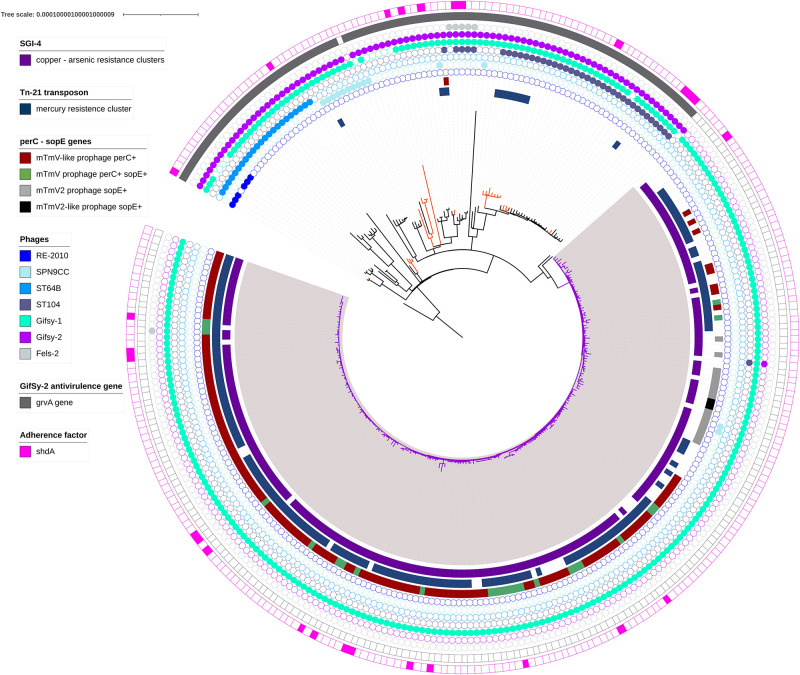
SNP core genome phylogenetic tree of 298 *S*. Typhimurium and *S*. 1,4,[5],12:i:- strains. The main clade A is indicated with purple branches. This group is composed by 90% of *S*. 1,4,[5],12:i:- strains. Orange branches indicate *S*. 1,4,[5],12:i:- strains not belonging to the main group. Strips illustrate the presence of the *Salmonella* genomic island 4 (SGI-4) carrying arsenic and copper resistance genes (purple strip), mercury resistance genes (*mer*TABCDE and *mer*R) in a Tn-21 like composite transposon (blue strip) and the mTmV, mTmV2 and mTmV-like phages (red, green, gray, and black strip). Presence/absence of phage is indicate with colored circles. The main differences observed of presence/absence of virulence factors are indicated – *grv*A and *shd*A genes – in gray and rose, respectively.

BLAST analysis of the mTmV sequence of the strain S04698-09 (accession number NZ_LN999997.1, nucleotides 5 022 794–5 037 238 and 1–24 715) against the 298 genomes revealed the presence of mTmV-like prophages in 175 genomes all of which were *S.* 1,4,[5],12:i:- ST34 belonging to the clade A with the exception of one strain belonging to the clade B (Figure 2 and Supplementary Table 5). Among these genomes, the presence of mTmV carrying both *sopE* and *perC* genes was detected in 25 genomes while mTmV-2, encoding only *sopE* was present in 15 genomes. All these 40 prophages harbored the site-specific integrase allowing integration in the *thrW locus*. 133 genomes contained other mTmV-associated prophages carrying only *perC* gene. These mTmV-like prophages do not encode the same integrase as mTmV and mTmV-2 suggesting a different integration site. To our knowledge, this is a first description of an element carrying only the *perC* gene in the *S*. 1,4,[5],12:i:- isolates in Europe. Further analyses are in progress to identify the insertion sites of this mTmV-like *perC* + prophage. Finally, two genomes carried only the *sopE* gene without the integrase sequence.

The two oldest strains carrying the mTmV prophage *per*C+ *sop*E+, 180911-18-6569 and ERR2003331, were isolated in 2002 in Germany from human sources. Interestingly, the strain 180911-18-6569 clustered with eight other strains isolated between 2002 and 2018 in Denmark, France, Germany, Italy, and the United Kingdom from humans, pigs, and pork (37 ± 8 SNPs within this group supported by 100% bootstrap values). The strain ERR2003331 clustered with eight strains isolated between 2002 and 2017 in Denmark, France and Germany from humans, cattles, duck meat and the environment (28 ± 9 SNPs within this group; supported by 64% bootstrap values) (Supplementary Figure 1).

The oldest strain carrying the *sopE* gene was S04698-09 isolated in 2009 in the United Kingdom from cattle. This strain was genetically related to two other strains isolated in the United Kingdom (L00211-09 and S01861-18) in 2012 and 2018 from horse and poultry, respectively (these 3 strains were genetically related with an average of 6 ± 3 SNPs). In France, the oldest stain (11CEB2326SAL) carrying this gene was isolated in 2011 from sheep’s milk. This strain was closely related to other six French strains and to two Germany strains (ERR2174791and ERR2174831) isolated in 2014 from pigs and beef, respectively (the seven French strains and the German ERR2174791 strain were separated by an average of 5 ± 8 SNPs). The first Italian and Danish strains carrying the *sopE* gene (ERR2382813 and ERR1540278) were isolated in 2012 and 2013 and clustered together with 12 other Italian and Danish strains isolated from humans, pigs, and pork (these 14 strains were separated by an average of 38 ± 6 SNPs). The three groups were supported by 100% bootstrap values.

The results obtained with the PHASTER software revealed the presence of seven intact prophages (Supplementary Table 6 and Figure 2). Gifsy-1, Gifsy-2, ST104, ST64B, SPN9CC, RE-2010, and Fels-2 were identified within 96% (286/298), 31% (92/298), 13% (39/298), 8% (23/298), 5% (14/298), 2% (6/298), and 2% (6/298) of the genomes, respectively. While Gifsy-1 was identified in all genomes belonging to the clade A, the other six prophages were almost exclusively identified in the strains of the clade B (Figure 2).

### Bayesian Analysis of Molecular Sequences

**FIGURE 3:**
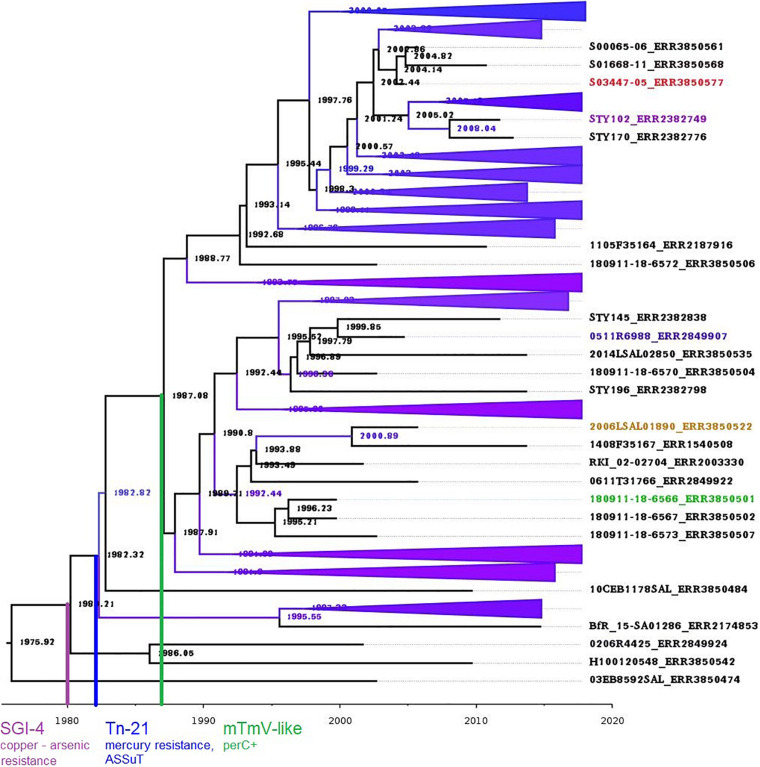
Bayesian analysis of the 205 genomes belonging to the clade A. Time-scale with the steps of evolution of *S*. 1,4,[5],12:i:- European clone ST34 is reported at the bottom. In green (DE), yellow (FR), blue (DK), purple (IT), and red (UK) the first *S*. 1,4,[5],12:i:- strains per country of our dataset.

Bayesian analysis was carried out for the dataset of the 205 *S*. 1,4,[5],12:i:- ST34 genomes of the clade A (Figure 3). The Kimura K3Pe+F+I model of nucleotide substitution was the best fit model obtained by iQtree program and was used in the time-scale phylogenetic analysis. The best Bayesian tree was obtained using relaxed Clock Log Normal with the Coalescent Exponential Population model. The true posterior was a log-normal distribution and the MCMC chain had length: 1000 million (Figure 3). Both, LSD and BEAST analyses indicated that the ancestral nodes of the *S.* 1,4,[5],12:i:-ST34 clone in Europe emerged in the year 1980.

The acquisition of the SGI-4 carrying ATPase efflux pumps for arsenic and copper resistance was pointed out to 1980 for this study subset (Figure 3). The initial insertion of the composite Tn21-like transposon in the *fljAB locus*, carrying the resistance to mercury, tetracycline, aminoglycosides, beta-lactames and sulfonamides was identified to have occurred two years later, in 1982 for the studied subset of genomes. A loss of the mercury resistance was observed in a subset of isolates around 1997 that still carried the Tn-21 – like transposon.

The analysis also revealed that independent events marked the acquisition of the phages mTmV and mTmV-2 within the five countries. The time scale phylogeny pointed out the acquisition of the putative mTmV prophage carrying *perC* gene (mTmV-like *per*C+) in 1987. Among our panel of strains, the mTmV prophage carrying *per*C gene was identified by a single event the first time in Germany in 2002 followed by the UK in 2005, Denmark in 2006, France in 2007, and Italy in 2012. This event was followed by the multiple independent acquisitions of mTmV prophage carrying both, the *per*C and the *sop*E genes (mTmV *perC*+, and *sopE*+) that occurred in the UK in 2009 in cattle sector. Finally, the mTmV2 prophage *sop*E+ occurred for the first time in the UK in 2010 in pig sector. However, majority of isolates with mTmV2 prophage carrying *sop*E gene were from Italy (Palma et al., 2018).

### Identification of Virulence Factors

A total of 73 different virulence factors were detected within the *S*. Typhimurium and *S*. 1,4,[5],12:i:- isolates in this study (summarized in the Supplementary Table 5). *Salmonella* Pathogenicity Islands (SPI)-1 and SPI-2 genes encoding type III protein secretion systems (T3SS) required for intestinal cells invasion were present in all genomes, as well as *sod*Cl gene implicated in survival in macrophage phagosome (Tidhar et al., 2015). Between 98 and 99% of strains carried *rat*B and *fim*CD genes involved in intestinal persistence and host cells adherence, respectively. The same percentage was observed for *bcf*A and *cgs*D genes, both involved in biofilm formation. The main differences between clade A and B were observed in presence/absence of *grv*A coding for an anti-virulence protein and *shd*A gene coding for an outer membrane protein implicated in adherence system. All genomes in the *S*. 1,4,[5],12:i:- clade A were free of *grv*A gene that is carried by Gifsy-2 phage (i.e., these genomes did not carry Gifsy-2 phage). Sixteen *shd*A positive strains were present in clade A (5%) and 11 in clade B (4%) (Figure 2).

The virulence factor genes (*iut*A, *iuc*D, *iuc*C, *iuc*B, and *iuc*A) involved in iron acquisition were identified in a single *S*. 1,4,[5],12:i:- strain (03CEB8592SAL) in the clade A. This strain was isolated in 2003 in France from turkey. BLAST analysis revealed that this virulence region was carried by a 15 Kb contig virtually identical (99.98% identity) to several plasmids from *E. coli* and other Enterobacteriaceae. The *E. coli* plasmid pACN001-B (KC853435.1) encoding these virulence factors has been previously characterized by Wang and collaborators (Wang et al., 2014).

**FIGURE 4:**
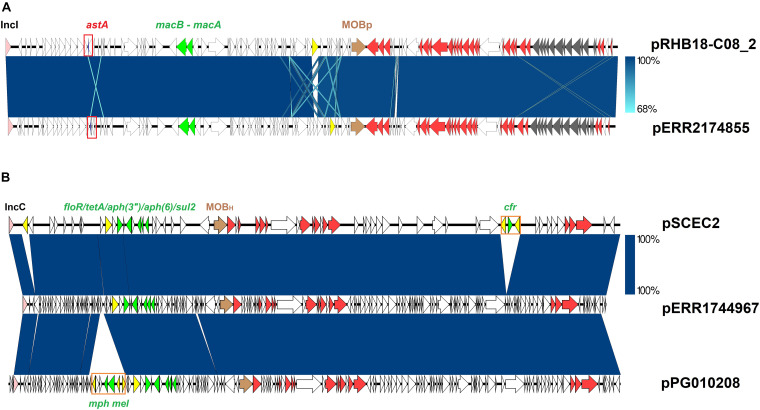
*Salmonella* plasmids previously identified in other Enterobacteria. Core plasmid sequences are colored pink (Inc replicon), brown (relaxase), and red (tra genes). Transposases and AMR determinants are represented with yellow and green arrows respectively. Blue areas between ORFs denote nucleotide sequence identity. **(A)** Sequence comparison of the *S. enterica* plasmid pERR2174855-2 with the *Escherichia fergusonii* plasmid pRHB18-C08_2 (CP055956.1). *E. coli* enterotoxin sequence is highlighted with a red square. Gray arrows represent genes coding for the formation of a pilus. **(B)** Sequence comparison of the *S. enterica* plasmid pERR1744967-1 with the *E. coli* plasmids pSCEC2 (KF152885) and pPG010208 (HQ023861).

The enterotoxin *ast*A was detected in a single *S*. 1,4,[5],12:i:- strain (ERR2174855) belonging to the clade A. This strain was isolated in 2015 in Germany from a bootswab sample taken from a laying hen flock. This virulence factor was located on a 116 Kb contig which shared 99% identity with a complete plasmid recovered from three strains of *E. fergusonii* (CP055956.1, CP057682.1 and CP055697.1) (Figure 4A).

The virulence plasmid (pSLT) encoding the pef, rck and spv operons was detected in 82 strains belonging mostly to the serovar Typhimurium (73%) and was absent in all isolates in clade A. Among these 82 strains, 63 encoded all pSLT loci while the rest were lacking one or more genes. Eight *S*. 1,4,[5],12:i:- of the clade B carrying pSLT did not encode the genes of the rck and pef operons and were lacking some *tra* genes from the mobilization system (Supplementary Table 7).

### Plasmidic and Genomic Acquired Resistance Genes

**FIGURE 5:**
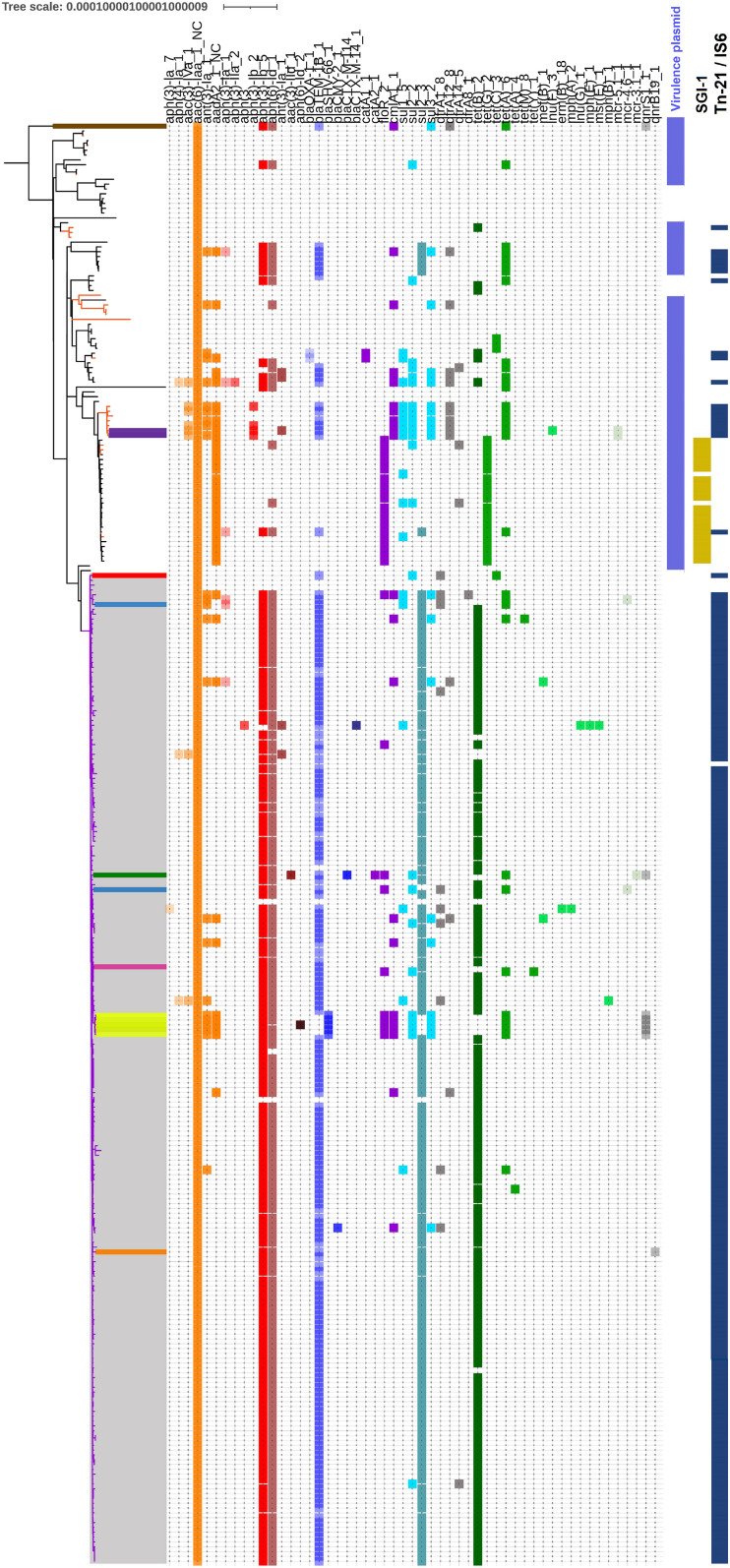
Distribution and diversity of acquired resistance genes. All the strains carrying plasmids are underlined with a colored range. The strains 2002LSAL10320 and 2004LSAL04350 harboring the *mcr*-5.1 gene mediating colistin resistance in a plasmid highly similar (99.95% of identity) to the *E. coli* plasmid pEC0674 (MF684783.1) are highlighted in purple. The strain ERR2849932 carrying the *qnr*S1 gene in a 28 Kb sequence similar to a fragment of the pK91-like plasmids is highlighted in brown. The strains ERR2174790 and ERR2003341 carrying the *mcr*-4.6 gene mediating for colistin resistance in a large contig (476 Kb) is highlighted in light blue. The strain ERR2187916 carrying the *mcr*-3.1 gene in a small contig identical to a fragment of the plasmid p08-5333.1 is highlighted in green. The strains ERR1744967-71 likely carrying 2 plasmids, the plncX-SHV encoding the *qnr*S1_1 gene and a *E. coli*-like plasmids never described before encoding the *floR*_2 gene, are highlighted in yellow and the strain ERR2849918 encoding the *qnr*B19 gene for quinolone in a 2.8 Kb contig with strong similarity (91–97% coverage) to small ColE-like resistance plasmid is highlighted in orange. The strain 03CEBSAL8592 carrying the plasmid pACN001-B encoding the *int*ABCD cluster for iron acquisition is in red. The strain ERR2174855 carrying a *E. fergusonii*-like plasmid never described before encoding the *ast*A gene for the enterotoxine A is in rose. The presence of the *Salmonella* virulence plasmid is signaled by a purple lilac column. SGI-1and Tn-21/IS6 acquisitions are also underlined with colored columns on the right.

Antibiotic resistance genes identified in all 298 genomes in this study are listed in Supplementary Table 8. As expected, all genomes harbored an *aac(6′)-Iaa_1_NC* gene (coding for a aminoglycoside *N*-acetyltransferase). Further most frequent genes were a*ph(6)-Id_1* (streptomycin phosphotransferase) and *aph(3′′)-Ib_5* (aminoglycoside *O*-phosphotransferase) present in 65 and 64% of genomes, respectively. *BlaTEM-1B_1*, *sul2_3* and *tet(B)_2* genes coding for resistance to beta-lactams, sulfonamides and tetracyclines were present in 65, 59, and 59% of strains, respectively (Figure 5). The genes *aph(3′′)-Ib_5*, *aph(6)-Id_1*, *blaTEM-1B_1*, *sul2_3* and *tet(B)_2*, conferring ASSuT profile, were simultaneously observed in 49% (146/298) of the genomes on either Tn-21 transposon (*n* = 138) or IS6 (*n* = 8) located in the *fljAB locus* (Figure 5 and Supplementary Table 2). This profile was the most frequent within the *S*. 1,4,[5],12:i:- strains and represented 71% (146/205) of genomes of the clade A (Figure 5).

#### Genes Mediating Phenicol Resistance

Among the 298 genomes, 54 carried genes mediating resistance to phenicols. The genes *floR_2, cmlA1_1, catA1_1* and *catA2_1* were found combined or alone. Sixteen of these 54 strains belonged to the clade A and 38 to the clade B (Supplementary Table 8).

The most frequent profile observed among the *S. Typhimurium* genomes of clade B carrying phenicol resistance was *aadA2_1_NC, tet(G)_2, aac(6′)-Iaa_1_NC* and *floR_2* genes mediating resistance to aminoglycosides, tetracyclines, beta-lactams and florfenicol, respectively. Interestingly, these four genes were carried by small contigs likely associated with a multiresistance bearing class1 integron region of the *Salmonella* Genomic Island-1 (SGI-1). The presence of this genomic island in the 21 *S. Typhimurium* genomes was confirmed by the presence of the SGI-1 integrase at the 3′-end of the *trm*E gene and the integron *int1*. The oldest strains with this profile were 03EB10285SAL, 03EB576SAL and 2003LSAL05838, three non-related strains isolated in 2003 in France from pork, sheep, and pig, respectively (Figures 1, 5).

Five *S*. 1,4,[5],12:i:- strains ERR1744967-ERR1744971 in the clade A, isolated in the United Kingdom from pigs in 2016, harbored five resistance genes (*sul2_2; aph(3′′)-Ib_5; aph(6)-Id_1; tet(A)_6; floR_2*) located on a 129 Kb contig (Figure 5 and Supplementary Figure 1). This genetic element displayed 99% nucleotide sequence identity to a family of IncA/C plasmids carrying different AMR determinants and distributed in several Enterobacteria (Fernandez Alarcon et al., 2011; Wyrsch et al., 2016). Sequence comparison of the plasmid isolated from ERR1744967 (p ERR1744967) with two *E. coli* plasmids (pSCEC2 and pPG010208) (Fernandez Alarcon et al., 2011; Zhang et al., 2014) confirmed the presence of a conserved backbone sequence and the acquisition of different resistance determinants (Figure 4B).

#### Genes Mediating Colistin Resistance

Three *mcr* genes (*mcr*-*3.1*, *mcr*-*4.6*, and *mcr*-*5.1*) encoding resistance to colistin were present in five of the 298 strains analyzed. The previously described *S*. 1,4,[5],12:i:- strain ERR2187916, isolated in 2011 in Denmark, contained the *mcr*-3.1 gene and 10 additional AMR genes (*aac(3)-IId_1, aph(3′′)-Ib_5, aph(6)-Id_1, blaCTX-M-55_1, blaTEM-1B_1, sul2_2, tet(A)_6, catA2_1, floR_2 and qnrS1_1*) in a small contig identical to a fragment of a larger plasmid, p08-5333.1 (CP039562.1), recovered from a *Salmonella enterica* isolated in Canada (Litrup et al., 2017; Pan et al., 2020).

Two *S*. 1,4,[5],12:i:- strains (ERR2003341 and ERR2174790) contained the *mcr*-4.6 gene on a plasmid virtually identical to the *S. enterica* plasmid pMCR_R3445 (MF543359.1) (Carattoli et al., 2017). However, further analysis of the contigs carrying this gene showed that the plasmid in ERR2003341 isolate was located inside a large contig of 476 Kb, suggesting an integration into the chromosome. These two strains were isolated in Germany from pigs in 2004 and 2014, respectively.

The last two *S. Typhimurium* strains isolated in France from cattle and pigs in 2002 and 2004, respectively (2002LSAL10320 and 2004LSAL04350) harbored the *mcr*-5.1 gene on a plasmid displaying 99.95% identity with previously described plasmids pSE11-03671 and pSE12-02284 isolated from two *S*. Typhimurium strains obtained from pigs and meat in Germany (Borowiak et al., 2019).

All five strains harboring *mcr* genes had between 11 and 14 AMR genes mediating resistance to six to eight antibiotic drug families (Figure 5 and Supplementary Table 8).

#### Genes Mediating Trimethoprim Resistance

Among the panel of the 298 strains, 30 strains (12 *S*. Typhimurium and 18 *S*. 1,4,[5],12:i:-) carried *dfr*A genes (*dfrA1_8, dfrA12_8, dfrA14_5, dfrA8_1*) mediating resistance to trimethoprim. Sixteen strains simultaneously carried AMR genes for aminoglycosides (i.e., gentamicin and streptomycin/spectinomycin), beta-lactamase, chloramphenicol, sulfonamides, tetracyclines, and trimethoprim. Of these 16 strains, 10 belonged to the clade B and 6 to the clade A. These strains were isolated between 2001 and 2018 in four countries (Denmark, France, Germany, and the United Kingdom), from human, pigs/pork, cattle, finfish, feed, and the environment. Even though this resistance profile is characteristic of the *S*. 1,4,[5],12:i:- Spanish clone, evidence of pUO-STmR/RV1-like plasmid (i.e., typically found in the Spanish clone) or insertion of IS26 in *flj*AB *locus*, presumably donated by pUO-STmR/RV1-like plasmids, were not observed (Figure 5 and Supplementary Table 8). Moreover, 15 different AMR gene profiles were observed for these 16 strains with only two *S*. 1,4,[5],12:i:- sharing the same AMR gene profile (2001LSAL02105 and 2005LSAL09168) (Supplementary Table 8).

#### Genes Mediating Quinolone Resistance

Seven strains carried *qnr* genes (*qnrB19* and *qnrS1*) mediating resistance to fluoroquinolone. A single *S*. 1,4,[5],12:i:- strain (ERR2849918), isolated in Denmark in 2018, was found to encode the *qnrB19* gene in a 2.8 Kb contig that exhibited a strong similarity (identical over 91–97% coverage) to a small ColE-like resistance plasmid identified in *S. enterica* and other Enterobacteriaceae (Karczmarczyk et al., 2010). The six other strains harbored the *qnrS1* gene. Interestingly, the *S.* Typhimurium strain ERR2849932, isolated in Denmark in 2018, carried *qnrS1* gene on a 28 Kb contig that was similar (55% coverage) to a part of the *qnrS1* harboring pK91-like plasmid previously described by Lima et al. (2019). Therefore, it is highly likely that this contig belongs to a plasmid - despite the fact that it did not carry any replicons or mobilization markers. This strain is characterized by the MLST profile ST313 more often associated with disseminated disease in sub-Saharan Africa commonly treated with antibiotics (Bawn et al., 2020). Five *S*. 1,4,[5],12:i:- strains (ERR1744967 to ERR1744971) carried the *qnrS1* gene on a 44 Kb contig, which showed high similarity (86% of coverage), with a fragment of the *Klebsiella* plasmid (pIncX-SHV) (Garcia-Fernandez et al., 2012). *In silico* typing revealed that this new putative plasmid encodes an IncX3 replicon type and MOBx. These five closely related strains (diversity of 10 ± 1 SNPs), all isolated in 2016 in the United Kingdom, were carrying two different multidrug resistance plasmids (pIncX-SHV and pSCEC2 like plasmids) with 11 AMR genes (i.e., *qnrS1, aac(6’)-Iaa, ant(3”)-Ia, aadA2, blaSHV-66, sul3, cmlA1* and *floR, sul2_2, aph(3”)-Ib, aph(6)-Id, tet(A)*) mediating resistance to five antibiotic drug families (aminoglycosides, beta-lactams, phenicols, sulfonamides, and tetracyclines).

## Discussion

Using the largest genome set of *S*. 1,4,[5],12:i:- from multiple European countries ever investigated, we unraveled the evolutionary history and main drivers of the global spread of the European ST34 ASSuT clone, which dominate *Salmonella* human infection in Europe and is repeatedly involved in food-borne outbreaks (European Food Safety Authority [EFSA], and European Centre for Disease Prevention and Control [ECDC], 2014, 2015, 2016, 2017, 2018, 2019; Bawn et al., 2020). Using the time-scaled phylogenies of the monophasic subset (Clade A) we estimated that the common ancestor of this *S*. 1,4,[5],12:i:- European clone emerged around 1980. This predicted date is consistent with the first *S*. 1,4,[5],12:i:- reported isolation from chicken carcasses in Portugal in 1986–1987 (Machado and Bernardo, 1990). This emergence was followed by a sharp increase in effective population size of the *S*. 1,4,[5],12:i:- ASSuT ST34 clone in the mid-1980s as reported by several publications (Switt et al., 2009; Wang et al., 2019; Sun et al., 2020). The results of Bayesian analysis point to ASSuT and heavy metals resistances as the main drivers of this expansion. The ampicillin, streptomycin/spectinomycin, sulfonamides, and tetracyclines resistance as well as mercury resistance were conferred by the insertion of a composite transposon of the Tn21-family in the *flj*AB *locus.* The arsenic and copper resistances were obtained by the acquisition of the conjugative element SGI-4 at the 3′ end of the phenylalanine tRNA-phe *locus*. Even though there were other *S.* Typhimurium and *S.* 1,4,[5],12:i:- clones with similar antimicrobial resistance circulating in Europe in 1980, the heavy metals resistance seems to have really determined the success of this clone in the late 1980s. The driving role of mercury resistance in the success of the clone is further supported by the fact that this compound had been widely used in herbicides and pesticides for seeds and grains production for animal feed since the last decades of the 19th century (Treccani, 2013) and it was not until 22 April, 1999 that the European parliament decided on the limitation of mercury in animal feed because of alarming observation about its accumulation in food chains (Jernelöv and Lann, 1971). Limits of mercury in feedstuffs introduced by Council Directive 99/29/EC are 0.1 mg/kg for all animals, except for pets where 0.4 mg/kg is tolerated.

On the other hand, the SGI-4 inserted in the tRNA-phe *locus* encodes genes involved in arsenic resistance and copper homeostasis. Copper compounds were authorized in EU as feed additives for all animal species and were regulated only from the 22 September, 2003 (Commission Implementing Regulation (EU) 2018/1039 of 23 July, 2018). The indirect use of heavy metals as herbicides or pesticides for the cultivation of seeds intended for animal feed (mercury) or the direct use of these substances as food supplements (copper) has allowed the accumulation of these heavy metals in farms where they exerted selective pressure on pathogens colonizing the intestinal tract of farm animals. Interestingly, even though arsenic is released into general environment as a result of human activity such as coal burning or burning of wood treated with arsenic-containing preservatives, the most important commercial compound, arsenic (III) oxide (also known as arsenic trioxide), is produced as a by-product in the smelting of copper (USGS data 2020). Without being a health problem, in Europe, the main sources of arsenic exposure for the general human population are particularly food, as grain-based processed products such as wheat bread, rice, milk and dairy products, and drinking water. Interestingly, according to EFSA data, the generic food group “Meat and meat products” possessed the highest levels of estimated inorganic arsenic (MB = 14.4 μg/kg) showing the accumulation of this compound in poultry, cattle, and pork (https://www.efsa.europa.eu/en/press/news/140306). Curiously, our results underline that the evolution of *S.* Typhimurium is driven by anthropogenic selection as recently observed by Bawn et al. (2020).

Blast analyses searching for *ars*BD, *cop*BCD, and *mer*C sequences in NCBI databases (i.e., available as of September 2020, data not shown) revealed that several *Salmonella* serovars (such as Newport, Saintpaul, Thompson, Kentucky, Senftenberg, and others) seem to also carry arsenical resistance operon/genes. In contrast, copper resistance operons/genes appeared to be less frequent but were nevertheless found in serovars Senftenberg, Anatum, Brandeburg, and Virchow. On the other hand, ATPase transporters for copper (CopA and GolT) and periplasmic cupredoxin detoxification systems encoded in the integrative and conjugative element SGI-4, were frequently found in other *Enterobacteriaceae* including *E. coli* (Rensing et al., 1999). Mercuric resistance was recently shown in the epidemic *Salmonella* serovar Derby clone ST40 responsible for 70% of human *S*. Derby infections in France (Sevellec et al., 2020). In *S.* Derby, the mercury resistance cluster (*mer*A, *mer*C, *mer*P, *mer*T, and *mer*R) is located on the transposon Tn-7 carried by the SGI-1 encoding also the *aadA2* and *sul1* genes mediating aminoglycosides and sulfonamides resistance (Sévellec et al., 2018). Further tests should be conducted to understand the possible transfer and the distribution of heavy metal resistance clusters between different *Salmonella* serovars and other enteric bacteria.

The second step of the European *S*. 1,4,[5],12:i:- ST34 clone evolution occurred in the 1990s (i.e., in 1987) with the acquisition of the ability to reduce nitrates in intestinal contents. Acquisition of mTmV-like phage *per*C+ is an expected key adaptive advantage for the spread of this clone exposed to other *Enterobacteriaceae* competition pressure. The ability to reduce nitrates in the host lumen by competing with the host’s microbiotic flora such as *E. coli*, is conferred by the acquisition of *per*C gene found on horizontally transferred elements like plasmids and prophages. The *per*C gene was initially identified in *E. coli* pEAF plasmid and its role recently elucidated. It controls the anaerobic metabolism by increasing the expression of genes necessary for nitrate reduction (Mellies et al., 2017). This gene was also detected in the genome of the epidemic *S*. Derby clone ST40 from France (unpublished data). LSD and Beast analyses pointed out a single event for this prophage acquisition in 1987.

The acquisition of mTmV prophage *per*C+ *sop*E+ was characterised by several independent events, with the first event dated in 1994. The sopE factor is involved in cellular invasion and disorganization of epithelial cell junctions, and therefore constitutes a key component of the infection process (Müller et al., 2009). Finally, the acquisition of the mTmV2 prophage *sop*E+ was also characterised by several independent events that have occurred over time, with the first event identified in 1995.

These results underlined that even if independent events led to the aquisiton of mTmV-like prophages carrying both *per*C+ and *sop*E+ genes, the pig and cattle sectors seem to have facilitated its acquisition and furthermore the commercial exchanges between countries enabled the spread of these *S*. 1,4,[5],12:i:- *per*C+ and/or *sop*E+ clones found all around Europe today.

The fact that the *sop*E gene was introduced by a mobile element such as a phage into the genome may explain why independent events have been observed. However, a Bayesian analysis focusing only on genomes with this gene could confirm the chronological sequence of events identified. Three identical copies of *sop*E were recently identified in the genome of the monophasic *S*. Typhimurium ST34 strain S01569-10 (Tassinari et al., 2020). Two chromosomal copies of *sop*E gene were located in distinct prophages, designated mTmV1569 and Ψm1569, and the third copy of the *sop*E gene was present in strain S01569-10 on an 81.1 Kb prophage plasmid, designated pS01569 (Tassinari et al., 2020). The nature of *sop*E gene identified in the genomes in this study is chromosomic, associated with both, mTmV and mTmV2 identified prophages. Analyses of the *sop*E gene sequences are in progress to better characterize this virulence factor and to determine the number of copies present par genomes.

Thus, even if epidemic *S.* 1,4,[5],12:i:- ST34 shared the same multi-drug resistance profile with other *S. Typhimurium* and monophasic clones circulating in Europe in 1980, its expansion was strongly related to the multiple-heavy metal resistance acquired by transposons, integrative and conjugative elements. Independent acquisition events of the mTmV phage occurred since 1990 in Europe, most likely facilitated by the proximity of this *Salmonella* with *E. coli* in pig and cattle sectors that boosted its spread. Finally but not less important, the expansion of this clone was facilitated by the escape of regulatory actions until 2011. The *S*. 1,4,[5],12:i:- was indeed included in the list of mandatory *Salmonella* in Europe only in late 2011 (CE n°1086/201121).

The genomic plasticity of this serovar is underlined in this study by the description of several *S*. 1,4,[5],12:i:- clones harboring different plasmids. In this study, iron uptake in the epidemic *S.* 1,4,[5],12:i:- ST34 strain 03CEB8592SAL was shown to be gained by an acquisition of the *E. coli* plasmid (pACN0061-B) (Wang et al., 2014). This acquisition indicates horizontal exchange of plasmids between *S*. 1,4,[5],12:i:- and different bacterial species. Other exchanges of genomic material with other Enterobacteria, such as *E. coli* and *Klebsiella*, were underlined by the presence of several plasmids carrying also genes for antibiotic resistance. Identification of the ColE-like plasmid, encoding the *qnrB19* gene conferring resistance to quinolones, which is present within our panel in the *S.* Typhimurium strain ERR2849918 isolated in Denmark in 2018 and previously identified in several *Enterobacteriaceae*, illustrates this concept very well. A second example is illustrated by the plasmids pSE11-03671 and pSE12-O2284 harboring *mcr-5.1* gene mediating colistin resistance in two *S.* 1,4,[5],12:i:- strains isolated in France from cattle and pigs between 2002 and 2004. As described by Borowiak et al. (2019), these two plasmids are highly similar to *E. coli* plasmid pEC0674 (MF684783.1) described by Hammerl et al. (2018) from porcine *E. coli* strains. The two *Salmonella* plasmids p-ERR1744967 and p-ERR2174855 identified in this study for the first time represent two other examples. The p-ERR1744967 shares 99% identity with two *E. coli* plasmids (pSCEC2 and pPG010208) (Zhang et al., 2014; Fernandez Alarcon et al., 2011) and harbors *qnrS1* gene mediating quinolone resistance in five epidemic *S*. 1,4,[5],12:i:- isolated in the United Kingdom in 2016. The p-ERR2174855 shares 99% identity with a plasmid isolated in three *E. fergusonni*. This plasmid was identified in an epidemic *S*. 1,4,[5],12:i:- isolated in Germany in 2015 and harbors *ast*A gene coding heat-stable enterotoxin. The gene *mcr*-*3.1* mediating resistance to colistin was identified in a strain isolated in 2011 in Denmark and carried by the plasmid p08-5333 already identified in *Salmonella* strains in Canada. This plasmid also carried 10 additional AMR genes conferring resistance to aminoglycosides, beta-lactams, sulfonamides, tetracyclines, florfenicol, and quinolone to this strain.

Finally, the gene *mcr*-*4.6* identified in two *S.* 1,4,[5],12:i:- were carried by sequence of plasmid (pMCR_R3445) located in large contigs suggesting likely its integration in the chromosome. The three *mcr* genes (*mcr-5.1, mcr*-*3.1*, and *mcr*-*4.6*) identified in this study were carried by five epidemic *S*. 1,4,[5],12:i:- strains isolated before 2016 (between 2002 and 2014) in Denmark, Germany, and France, from pigs and cattle. Interestingly, the study recently published by Biswas et al. (2019) shows that all the colistin-resistant *S.* 1,4,[5],12:i:- ST34 harboring *mcr*-*3.1* gene described in the literature are related to the first conjugative plasmid carrying this gene described in *E. coli* (Yin et al., 2017), indicating Southeast Asia as the potential reservoir for their global dissemination. Indeed, all these *S.* 1,4,[5],12:i:- ST34 *mcr*-*3.1*+ clones carried multiple antibiotic resistance genes (Biswas et al., 2019).

In Europe, while colistin had long been excluded from the panel of antibiotics used in human medicine because of its renal toxicity, fluoroquinolones are used in humans. Colistin has been indeed regularly used in veterinary medicine, notably for the treatment of colibacillosis infections in animals (El-Sayed Ahmed et al., 2020). Since 2016, colistin use is regulated by the European Medicines Agency (EMA). With regard of the discovery of *mcr* genes (Bialvaei and Samadi Kafil, 2015), it has been agreed to reduce the use of colistin in food-producing animals in Europe by 65% (Madec, 2017). The choices concerning the use of antibiotics are based on delicate balances. A complete ban on the use of colistin in veterinary medicine could have increased the pressure on other critically important antibiotics (i.e., quinolones and cephalosporins). To reduce the use of colistin and other antibiotics in food producing species, the use of heavy metals in feed has been promoted, such as zinc oxide in piglets already before 2013 (ANSES, 2013). A gene encoding resistance to this heavy metal is likely to be found in *Salmonella* in the coming years, as is already the case for *E. coli* (Ghazisaeedi et al., 2020; Yang et al., 2020).

In conclusion, to our knowledge this is the first comparison of a large panel of *S.* 1,4,[5],12:i:- strains isolated from several European countries. Our results allowed the characterization of the ancestral *S.* Typhimurium that gave birth to the epidemic European *S.* 1,4,[5],12:i:- clone ST34 in the 1980s. This ancestor, like the *S*. Typhimurium strain ERR1540279 at the basal node harboring epidemic *S*. 1,4,[5],12:i:- ST34, possessed *bcf*A and *cgs*D genes involved in biofilms formation and the SodCl factor that allows the survival of *Salmonella* in the phagosome of host macrophages. This ancestor was lacking the Gifsy-2 phage harboring *grv*A gene coding for a factor known to affect the pathogenicity of *Salmonella* in the host by decreasing its resistance to toxic oxygen species present in the host lumen (Ho and Slauch, 2001). Furthermore, it did not have the pSLT virulence plasmid and the outer membrane protein Rck that is involved in the invasion of phagocytic and non-phagocytic host cells during host cell invasion (Mambu et al., 2017). The acquisition of the SGI-4 in 1980 and the integration of the Tn21 transposon in 1982 marked the emergence of the European epidemic clone in the year 1980s with the ability to resist copper, arsenic and mercury toxicity and several antibiotic classes used in food-producing animals. Subsequently, in 1990s exchanges of genetic material with other Enterobacteria that colonize the intestinal lumen of farm animals provided the genetic background for the acquisition of the ability to reduce nitrates in the host lumen (*per*C acquisition) and to facilitate the disorganization of epithelial cell junctions (*sop*E acquisition) (mTmV and mTmV2 prophages). Acquisition of different plasmids allowed the rising of different clones with multi drugs resistance to antibiotic families such as colistin and quinolone. The epidemic monophasic *Typhimurium* ST34 clones are circulating in Europe and are present in all sectors from humans to farm and environment, but remain predominantly associated with the pig reservoir and does not persist long-term at individual farm level in other food animal species (Gosling et al., 2018).

## Data Availability Statement

The datasets presented in this study can be found in online repositories. The names of the repository/repositories and accession number(s) can be found in the article/Supplementary Material.

## Author Contributions

SC-S, P-ED, M-YM, and LP conceived the study. SC-S, EC, P-ED, YT, and LP contributed equally to the design and analysis of data. AF and PB conceptualized algorithms. SC-S, EL, SB, SS, FP, MG, and LP obtained, selected, and provided clinical strains. SC-S drafted the manuscript. P-ED, SB, SS, and LP reviewed the report. All authors commented and approved the final manuscript, took public responsibility for appropriate portions of the content, and agreed to be accountable for all aspects of the work in terms of accuracy or integrity.

## Conflict of Interest

The authors declare that the research was conducted in the absence of any commercial or financial relationships that could be construed as a potential conflict of interest.

## Footnotes

1 https://www.genomicepidemiology.org/
