# Supplementary figures and images for: The Spatiotemporal Dynamics and Microevolution Events That Favored the Success of the Highly Clonal Multidrug-Resistant Monophasic Salmonella Typhimurium Circulating in Europe
Source: Front Microbiol. 2021 May 21;12:651124. doi: 10.3389/fmicb.2021.651124 (PMC8175864; doi:10.3389/fmicb.2021.651124)

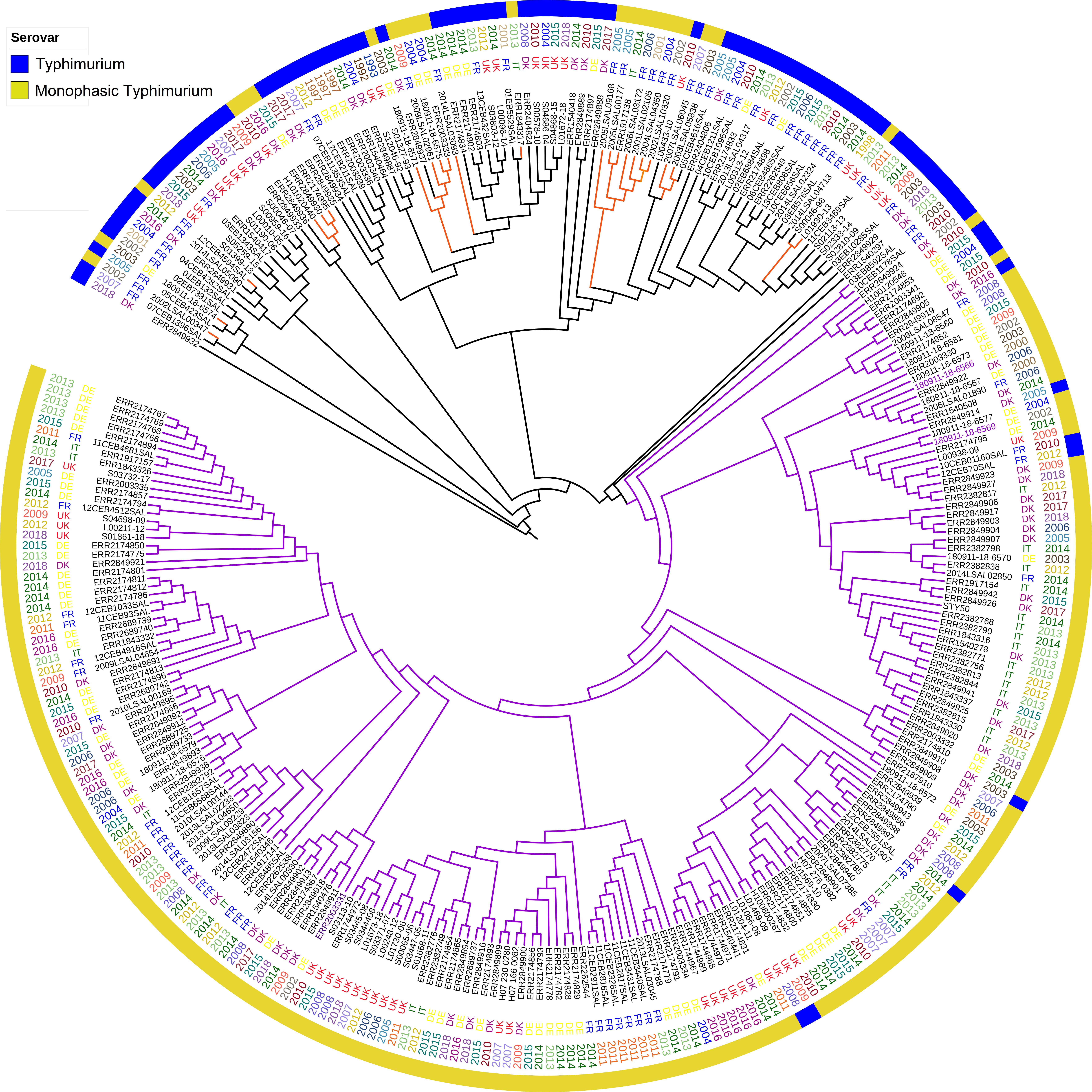

Supplement: Supplementary file 1 [file Image_1.JPEG]
